# Oxidized phospholipids and lipoprotein-associated phospholipase A_2_ as important determinants of Lp(a) functionality and pathophysiological role

**DOI:** 10.7555/JBR.31.20160009

**Published:** 2018-01-26

**Authors:** Alexandros D. Tselepis

**Affiliations:** Atherothrombosis Research Centre / Laboratory of Biochemistry, Department of Chemistry, University of Ioannina, 45110 Ioannina, Greece.

**Keywords:** atherosclerosis, calcific aortic valve stenosis, coronary artery disease, lipoprotein (a), lipoprotein-associated phospholipase A_2_, oxidized phospholipids

## Abstract

Lipoprotein(a) [Lp(a)] is composed of a low density lipoprotein (LDL)-like particle to which apolipoprotein (a) [apo(a)] is linked by a single disulfide bridge. Lp(a) is considered a causal risk factor for ischemic cardiovascular disease (CVD) and calcific aortic valve stenosis (CAVS). The evidence for a causal role of Lp(a) in CVD and CAVS is based on data from large epidemiological databases, mendelian randomization studies, and genome-wide association studies. Despite the well-established role of Lp(a) as a causal risk factor for CVD and CAVS, the underlying mechanisms are not well understood. A key role in the Lp(a) functionality may be played by its oxidized phospholipids (OxPL) content. Importantly, most of circulating OxPL are associated with Lp(a); however, the underlying mechanisms leading to this preferential sequestration of OxPL on Lp(a) over the other lipoproteins, are mostly unknown. Several studies support the hypothesis that the risk of Lp(a) is primarily driven by its OxPL content. An important role in Lp(a) functionality may be played by the lipoprotein-associated phospholipase A_2_ (Lp-PLA_2_), an enzyme that catalyzes the degradation of OxPL and is bound to plasma lipoproteins including Lp(a). The present review article discusses new data on the pathophysiological role of Lp(a) and particularly focuses on the functional role of OxPL and Lp-PLA_2_ associated with Lp(a).

## Introduction

Lipoprotein(a) [Lp(a)] consists of a low-density lipo-protein (LDL)-like particle to which a large, highly gly-cosylated apolipoprotein(a) [apo(a)] is covalently bound to the apoB-100 moiety of LDL *via* a single disulfide bridge^[1– 3]^. Apo(a) is highly homologous to the plasma protease zymogen, plasminogen, which contains five tri-loop structures stabilized by three disulfide bonds, named as kringles (K), and a protease domain; thus, it can be activated to plasmin. Apo(a) contains only KIV and KV and has an inactive protease-like domain^[1- 5]^. This domain is catalytically inactive, despite having an intact Ser-His-Asp catalytic triad^[6]^. A Ser^561^-Ile^562^ substitution for Arg^561^-Val^562^ has been proposed to render the protease-like domain in apo(a) inactive^[6, 7]^. Importantly, apo(a) contains 10 subtypes of KIV (KIV-1 to KIV-10); the KIV-2 subtype being present in variable numbers (5 to 50) of identically-repeated copies. KIV-9 contains an additional cysteine residue (Cys^4057^), which is attached by a disulfide bond to a cysteine residue (Cys^4326^) of apoB-100, located near the binding site of LDL to its receptor^[8- 10]^. Apo(a) is highly polymorphic in length due to the number variation of the KIV-2 copies; thus, the molecular mass of apo(a) isoforms can range between 200 and 800 kDa^[1]^. Apo(a) is encoded by the *LPA* gene, which contains a 5.6 kb segment existing in multiple repeats (KIV-2 repeat pol-ymorphism) that is responsible for the apo(a) isoform variation^[7]^. Plasma Lp(a) levels vary widely among individuals, are inversely correlated with the apo(a) size and are primarily genetically determined by varia-tion in the *LPA* gene coding for apo(a)^[11]^.

Lp(a) is considered a causal risk factor for ischemic cardiovascular disease (CVD)^[12, 13]^. The evidence for a causal role of Lp(a) in CVD is based on data from large epidemiological databases, mendelian randomization studies, and genome-wide association studies linking genetically determined Lp(a) levels to CVD events. In this regard, epidemiological studies suggest a log-linear relationship between circulating Lp(a) levels and CVD risk that is independent of other lipid measures and con-ventional risk factors. Mendelian randomization stud-ies demonstrate a causal, multivariable-adjusted, linear association between genetically determined Lp(a) lev-els and CVD risk. Finally, genome-wide association studies show that the *LPA* variants are strongly asso-ciated with reduced copy numbers of KIV-2 repeats, increased Lp(a) levels, and higher CVD risk^[12- 13]^. Despite the well-established role of Lp(a) as a causal risk factor for ischemic CVD, the underlying mecha-nisms by which it mediates atherogenicity are not well understood^[12]^. The present review article focuses on the pathophysiological role of Lp(a) and particularly dis-cusses new data on the potential role of two Lp(a) com-ponents, the oxidized phospholipids (OxPL) and the lipoprotein-associated phospholipase A_2_ (Lp-PLA_2_), in its functionality.

### Oxidized phospholipids: Formation and association with Lp(a)

A key role in the Lp(a) functionality may be played by its OxPL content^[14]^. OxPL are generated by the oxi-dation of polyunsaturated fatty acid residues, which are usually esterified at the *sn*-2 position of phospho-lipids^[15- 17]^. Oxidation of such phospholipids is initiated either enzymatically by lipoxygenases or by reac-tive oxygen species and propagates *via* the classical mechanism of lipid peroxidation chain reaction. This implies that the production of OxPL cannot be regu-lated by adjusting the amount or activity of enzymes; hence, there is an uncontrolled generation of OxPL during oxidative stress^[15- 17]^. OxPL can be formed on cell membranes under oxidative stress conditions or during apoptosis and cell death or on LDL during its oxidative modification. The major bioactive lipids in oxidatively modified LDL (OxLDL) are derived from oxidation of sn-2 arachidonoyl phospholipids^[18- 19]^. Three bioactive OxPL derived from oxidation of 1-palmitoyl- 2-arachidonoyl-sn-glycero- 3-phosphoryl-choline have been primarily identified as 1-palmitoyl-2-oxovaleroyl-sn-glycero-3-phosphorylcholine (POVPC), 1-palmitoyl-2-glutaroyl-sn-glycero-3-phosphoryl­ choline (PGPC), and 1-palmitoyl-2-(5,6- epoxyisopros-tane E2)-sn-glycero-3-phosphorylcholine (PEIPC)^[20- 21]^. POVPC and PGPC are typically present in OxLDL, can induce various cellular responses or cell death and have been detected in atherosclerotic lesions^[20- 21]^.

By using the murine monoclonal antibody E06, an IgM natural antibody that specifically binds to the phosphorylcholine (PC) head group of oxidized but not native phospholipids, it was possible to detect the con-tent of PC-OxPL per apoB-100 particle (OxPL/ApoB) in plasma and lipoprotein subspecies^[22- 23]^. E06 recog-nizes several PC-OxPL molecules, primarily POVPC, but not PGPC^[24]^. Importantly, most of circulating OxPL/ApoB are associated with Lp(a) with only small amounts found on LDL and high-density lipoprotein (HDL)^[25]^. Although the underlying mechanisms lead-ing to this preferential sequestration of OxPL on Lp(a) over the other lipoproteins are mostly unknown, *in vitro* experiments demonstrated that the more polar OxPL can exchange from the OxLDL donor to the Lp(a) particle in a manner independent on the lipid transfer proteins, which usually mediate the transfer of lipids among the lipoprotein particles in plasma^[25]^. In support of this hypothesis, it has been reported that immediately after iatrogenic plaque rupture due to percutaneous cor-onary intervention (PCI), plasma levels of Lp(a) and OxPL/apoB increased by 65% and 35%, respectively, the 50% of OxPL detected in plasma being associated with Lp(a), whereas the other 50% were detected on other apoB lipoproteins. However, by 6h, nearly all of the OxPL were associated with Lp(a)^[23]^. Lp(a) contains OxPL, such as POVPC, which are recognized by E06, as well as other OxPL molecules such as PGPC, which are not recognized by E06^[24- 25]^. These results suggest that the ability of Lp(a) to bind various OxPL is a gen-eralized property of this lipoprotein and it is not lim-ited only to E06-recognized OxPL^[25]^. A proportion of Lp(a)-associated OxPL are covalently bound to apo(a), primarily to its KIV10 domain. In general, candidate amino acids for OxPL binding are cysteines, lysines, and histidines. However, all six cysteines are occupied in disulfide bonds and there are no lysines in KIV10 of human apo(a). Therefore, it appears that the 3 his-tidines present in KIV10, are likely the sites in KIV10 that bind OxPL. In particular, His^31^ and His^33^, which flank Arg^32^, are strong candidates for OxPL binding^[26]^. A role in the association of OxPL with apo(a) may be also played by β-2 glycoprotein I (β_2_GPI), which can bind to apo(a)^[27]^, as well as to anionic phospholipids and OxPL^[28- 29]^. However the contribution of β_2_GPI-mediated bridging of OxPL and apo(a) in the overall association of OxPL with apo(a) needs to be further established^[30]^. OxPL do not only bind to apo(a) but are also present in the lipid phase of Lp(a)^[25]^. The factors that determine the distribution of OxPL between apo(a) and lipid phase of Lp(a), the biological meaning of this distribution in terms of Lp(a) functionality and patho-physiological role as well as possible variations in the relative amounts of OxPL on apo(a) versus the lipid phase of Lp(a) in various disease states, remains to be elucidated. In this regard, it was demonstrated that the amount of OxPL/ApoB in organic extracts of human Lp(a) varied considerably, with about 30%–70% of them in various subjects being lipid soluble^[25]^.

### Role of OxPL on Lp(a)-mediated cardiovascular risk

Several studies *in vitro* have demonstrated that OxPL interact with specific binding sites and various signal transduction receptors as well as pattern recognition receptors present on the cell surface, including CD36, SRB1, EP2, VEGFR2 and the platelet activating factor (PAF) receptor^[16, 31]^. Interaction of distinct OxPL species with the above receptors leads to the activation of indi-vidual signaling pathways in various cell types. Many cellular events are initiated and modulated by biologi-cally active OxPL such as the binding of leukocytes to endothelial cells, upregulation of expression, produc-tion and secretion of various cytokines and chemokines *in vitro *and in animal models* ex vivo *and* in vivo*^[32- 38]^,**modulation of expression of a number of genes related to atherosclerosis, angiogenesis, inflammation and wound healing in various cell types *in vitro*^[39- 40]^. OxPL also play important role in the recognition and uptake of OxLDL by macrophages. Increased levels of OxPL have been detected in apoptotic cells *in vitro*^[41- 42]^ and cells stimulated with inflammatory agonists *in vitro*^[32]^, as well as in various organs and tissues under patholog-ical conditions, including plasma of patients with cor-onary artery disease^[14]^, and advanced atherosclerotic lesions *in vitro*^[43- 44]^ in animal models *in vivo*^[21, 44]^. Upon formation, OxPL are mainly associated with Lp(a) *invivo *and several studies support the hypothesis that the risk of Lp(a) is primarily driven by its OxPL content^[45]^. Lp(a) at very high plasma levels promotes atheroscle-rosis and has been hypothesized to exert antifibrino-lytic / prothrombotic activities and to contribute to wound healing. Epidemiological studies have shown a remarkable correlation of plasma levels of OxPL/apoB and Lp(a)^[45- 47]^. Importantly, OxPL are mainly present on small apo(a) isoforms and their plasma levels are strongly correlated with Lp(a) particles having the low-est number of kringle repeats, this correlation being weakest for the largest Lp(a) isoforms^[45- 47]^. The stronger association of oxPL with small Lp(a) isoforms may at least partially explain their enhanced atherogenicity as well as their association with higher CVD risk as com-pared with large ones^[46- 48]^. High plasma OxPL/apoB levels independently predict the presence and extent of angiographically determined coronary artery disease, OxPL levels also identify the presence and progres-sion of carotid and femoral atherosclerosis, and pre-dict CVD events over a 10 year interval^[45- 47]^. Recent studies have demonstrated that Lp(a) may also play a causal role in the pathophysiology of calcific aortic valve stenosis (CAVS), a chronic disorder characterized by pathological mineralization and remodeling^[49]^. A genome-wide association study has identified a genetic variant (rs10455872) in the *LPA* gene locus, determin-ing plasma levels of Lp(a), to be causally related to CAVS^[50]^. A recent prospective study involving 77,680 persons (from 2 large prospective studies of the Danish general population, the CCHS; Copenhagen City Heart Study and the CGPS; Copenhagen General Population Study), demonstrated that increased Lp(a) levels and corresponding 3 *LPA* genetic variants (all associated with Lp(a) levels) were associated with increased risk of aortic stenosis in the general population, with lev-els>90 mg/dL predicting a threefold increased risk^[51]^. The results of these studies suggest that the association between Lp(a) and aortic stenosis may be causal^[50- 51]^. Importantly, the recent ASTRONOMER (Aortic Stenosis Progression Observation: Measuring Effects of Rosuvastatin) trial analysis demonstrated that ele-vated OxPL/apoB and Lp(a) levels are independently associated with an increased risk of echocardiograph-ically determined aortic stenosis progression rate^[52]^. Furthermore, this faster progression rate translated to a higher need for aortic valve replacement, which was accentuated in younger patients with elevated OxPL/ apoB or Lp(a) levels^[52]^. The risk of Lp(a) as an inde-pendent predictor of the progression of aortic stenosis could be explained by OxPL/apoB or OxPL/apo(a) lev-els. These findings support the hypothesis that aortic stenosis progression and need for aortic valve replace-ment are mediated by the OxPL content of Lp(a)^[52- 53]^. Overall, the results of the above studies provide strong evidence that Lp(a) and its OxPL content may mediate a common biological influence on atherosclerosis and CVD as well as on CAVS.

### Lipoprotein-associated phospholipase A_2_: enzymatic properties and binding to lipoproteins

Detoxification of reactive OxPL comprises the mechanisms that terminate peroxidation chain reaction and inactivate chemically reactive toxic groups pro-duced by oxidation^[54]^. OxPL subspecies can also be detoxified through enzymatic degradation catalyzed by several enzymes such as glutathione peroxidases^[55]^ and aldo-keto reductases^[56]^. An important enzyme that cata-lyzes the degradation of OxPL is lipoprotein-associated phospholipase A_2_ (Lp-PLA_2_). Lp-PLA_2_, also named as platelet-activating factor (PAF)-acetylhydrolase, exhib-its a Ca^2+^-independent phospholipase A_2_ activity and catalyzes the hydrolysis of the ester bond at the *sn-2* position of PAF and OxPL ^[57]^. OxPL are hydrolyzed by Lp-PLA_2_ into oxidized free fatty acid (OxFFA) and lysophosphatidylcholine (lyso-PC)^[57]^. Like OxPL, both products of Lp-PLA_2_ activity manifest pro-­ inflammatory and proatherogenic effects. However, it has not been established which is more clinically rele-vant^[57- 58]^. Lp-PLA_2_ circulates in plasma in active form, the vast majority of plasma enzyme being associated with LDL while a smaller amount is associated with high-­ density lipoprotein (HDL)^[57- 58]^. Lp-PLA_2_ is also bound to Lp(a)^[59]^. Interestingly, it was demonstrated that Lp(a) is enriched in Lp-PLA_2_ since it contains 1.5 to 2-folds higher enzyme mass and a 7-fold higher specific activity compared with LDL when assayed at equimolar protein concentrations^[30, 60]^. The binding of Lp-PLA_2_ on LDL is primarily mediated through the enzyme α-helix (114-126). Particularly, residues Trp-115, Leu-116 and Tyr-205 of Lp-PLA_2_, and to lesser extent Met-117, are critical for enzyme association with LDL^[61]^. Lp-PLA_2_ is bound primarily on apoB-100 and especially on its carboxyl terminus (residues 4119– 4279)^[61]^. The binding of Lp-PLA_2_ on HDL is mediated through its C-terminal residues His-367, Met-368, Leu-369, Lys-370^[62]^. Met-368 and Leu-369 residues play a prominent role for this binding, whereas a moderate contribution may have His-367 and Lys-370 residues. Met-368 and Leu-369 are necessary, but not sufficient for binding to HDL, suggesting that His-367 and Lys-370 either directly participate in Lp-PLA_2_ association with HDL or contribute to the formation of a binding pocket that optimizes interaction of Met-368 and Leu-369 with the lipoprotein^[62]^. Furthermore, it has been demonstrated that regions 107–120, 192–204 and 360– 368 of Lp-PLA_2_ contribute to enzyme association with HDL, the region 192–204 being particularly important for Lp-PLA_2_ interaction with apoA-I^[63]^. The major role in the attachment of Lp-PLA_2_ on Lp(a) is played by its apoB-100 moiety, whereas the enzyme does not bind to apo(a)^[59- 60]^. Importantly, there are marked differences in the enzyme catalytic properties among the various Lp(a) isoforms, the small isoforms exhibiting higher apparent Km values, ie being less active compared to large ones, suggesting that the apo(a) may influence the association of Lp-PLA_2_ with Lp(a), although it does not bind the enzyme itself^[59]^. The factors that contrib-ute to the differences among the small and high Lp(a) isoforms in the enzyme catalytic efficiency remain still unknown. However, the low catalytic efficiency of the Lp-PLA_2_ associated with the small apo(a) isoforms could be one of the factors that favor the sequestration of plasma oxPL on these isoforms^[30, 59]^, and conse-quently the strong correlation between small Lp(a) iso-forms and oxPL/apoB levels in plasma^[45- 47]^. However other, unknown yet, factors may contribute to this phe-nomenon, which needs to be further investigated.

### Lipoprotein-associated phospholipase A_2_ as an important determinant of Lp(a) functionality

**Fig.1:**
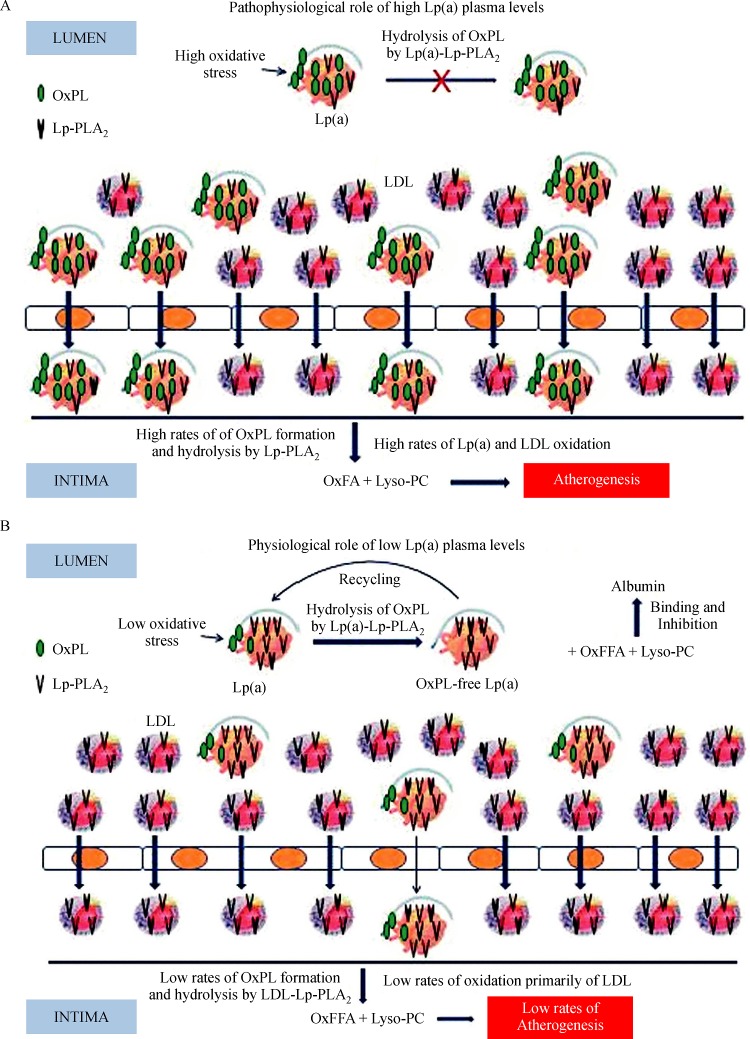
**Roles of different concentrations of Lp(a)-Lp-PLA**_**2**_**in plasma. **

Due to degradation and inactivation of the pro-­ inflammatory phospholipid PAF, initial data supported an anti-inflammatory role of Lp-PLA_2_ (***Table 1***)^[57]^. However, the concept on the pathophysiological role of Lp-PLA_2_ significantly changed when it was demon-strated that Lp-PLA_2_ also hydrolyzes OxPL into OxFFA and lyso-PC. These products are accumulated in the artery wall and display a wide range of pro-­inflammatory, proapoptotic and proatherogenic effects^[57]^. More recent studies provided evidence that the role of plasma Lp-PLA_2_ in atherosclerosis may depend on the type of lipoprotein particle with which this enzyme is associ-ated^[57- 58]^. In this regard, anti-inflammatory and antiath-erogenic effects of the LDL-Lp-PLA_2_ attributed to the catabolism and inactivation of PAF and OxPL mole-cules exhibiting pro-­ inflammatory and proatherogenic activities have been described in ***Table 1***^[57- 58]^. In addi-tion, pro-­ inflammatory and proatherogenic effects of the LDL-Lp-PLA_2_ attributed to the hydrolysis of OxPL and generation of OxFFA and lyso-PC have been described in ***Table 1***^[57- 58]^. LDL-Lp-PLA_2_ is increased in patients with primary hypercholesterolemia and combined hyperlipidemia and data from large Caucasian popu-lation studies have supported plasma Lp-PLA_2_ (pri-marily LDL-associated Lp-PLA_2_) as a cardiovascular risk marker, independent of and additive to traditional risk factors (***Table 1***)^[64- 65]^. On the contrary, the HDL-associated Lp-PLA_2_ may express anti-inflammatory, antioxidative and antiatherogenic activities. It also enhances the HDL-induced cholesterol efflux *in vitro *(***Table 1***)^[58, 65]^. HDL-Lp-PLA_2_ is reduced in patients with combined hyperlipidemia, primary hypertriglyc-eridemia, pre-diabetes and metabolic syndrome^[58, 65]^ and is also independently associated with lower risk for cardiac death (***Table 1***)^[66]^. The role of Lp(a)-associated Lp-PLA_2_ (Lp(a)-Lp-PLA_2_) has not been adequately studied. In subjects exhibiting high Lp(a) plasma lev-els, the Lp(a)-Lp-PLA_2_ may play a role similar to that observed for the LDL-Lp-PLA_2_ in the artery wall. Lp(a) can be accumulated preferentially to LDL within lesions to an extent proportional to its plasma levels where it binds very tightly to lesion components^[67, 68]^. In the intima, Lp(a) may transfer preformed OxPL, and it can undergo oxidative modification (***Fig. 1A***) thus further enriched in OxPL^[59]^. Lp(a)-bound OxPL are then hydro-lyzed by the Lp(a)-Lp-PLA_2_ to OxFFA and lyso-PC^[59]^, which significantly contribute to plaque formation^[57, 69]^. Consequently, through this mechanism, the Lp(a)-Lp-PLA_2_ may significantly influence the biological activities of oxidized Lp(a) in the artery wall^[70, 71]^, thus promoting atherogenesis (***Fig. 1A***)^[17, 58]^.

**Tab.1:** Physiological and pathophysiological functions of Lp-PLA_2_**associated with different lipoprotein particles

Lp-PLA_2_			
lipoprotein carrier	Physiological functions	Pathophysiological functions	Clinical evaluation
LDL-Lp-PLA_2_	Antiinflammatory and antiatherogenic through degradation of PAF and pro-inflammatory OxPL	Pro-inflammatory and proatherogenic through generation of OxFFA and lyso-PC	Increased levels in primary hypercholesterolemia and combined hyperlipidemia Independent marker of cardiovascular risk
HDL-Lp-PLA_2_	Antiinflammatory, Antioxidative Enhancement of HDL-induced cholesterol efflux Antiatherogenic		Reduced levels in combined hyperlipidemia, primary hypertriglyceridemia, pre-diabetes, metabolic syndrome
Lp(a)-Lp-PLA_2_	Antiinflammatory		Association with lower risk for cardiac death
low levels	Antiatherogenic		
Lp(a)-Lp-PLA_2 _high levels		Pro-inflammatory Proatherogenic	Low catalytic efficiency in patients with coronary artery disease

Abbrevations: Lp-PLA_2_; Lipoprotein-associated phospholipase A_2_, LDL; low density lipoprotein, Lyso-PC; lysophosphatidylcholine, HDL; high density lipoprotein, Lp(a); lipoprotein (a), OxFFA; oxidized free fatty acid.

In contrast to the above pathophysiological role of Lp(a)-Lp-PLA_2_ in the artery wall, this enzyme may confer Lp(a) with a physiological role in the circulation. Indeed, in contrast to the other plasma lipoproteins for which the physiological role in lipid metabolism has been well established, the physiological role of Lp(a) is still unknown. The existence of Lp-PLA_2_ on Lp(a) in concert with the preferential accumulation of OxPL on circulating Lp(a) may confer this lipoprotein with a physiological role as a scavenger of oxPL in human plasma^[72]^. Thus, under normal conditions (low oxida-tive stress, absence or low inflammation), Lp(a) may be beneficial by collecting OxPL from various sources, which are then degraded by Lp(a)-Lp-PLA_2_. Under these conditions, the pro-inflammatory lyso-PC, deriv-ing from the degradation of OxPL, could then be trans-ferred in the circulation from Lp(a) to albumin, which represents the major carrier and inhibitor of lyso-PC in plasma (***Fig. 1B***)^[73]^. Importantly, we had previ-ously shown that the Lp(a) of patients with coronary artery disease carries significantly lower amounts of Lp-PLA_2_ mass that expresses lower catalytic efficiency compared with controls, a phenomenon which is not observed for the LDL-Lp-PLA_2_^[30]^. The low catalytic efficiency of the Lp(a)-Lp-PLA_2_ in these patients may be due to the sequestration of oxPL on the apo(a) moi-ety of Lp(a) and may represent an important defect of Lp(a) in these patients since it exhibits a diminished capability to degrade and detoxify OxPL (***Fig. 1A***)^[30]^.

Overall, the OxPL and Lp-PLA_2_ components of Lp(a) are important determinants of its functionality. However, this Lp(a) functionality may be influenced by various factors such as the Lp(a) plasma levels, the extent of OxPL production (low or high oxida-tive stress, inflammation, apoptosis, tissue injury) as well as the levels of Lp(a)-Lp-PLA_2_ activity. In this regard, it should be pointed out that despite the con-sistent findings of many clinical trials showing that the total plasma Lp-PLA_2_, which represents primarily the LDL-associated enzyme, is an independent risk factor for cardiovascular disease, the results of two recent phase 3 clinical trials (STABILITY; Stabilization of Atherosclerotic Plaque by Initiation of Darapladib Therapy, and SOLID-TIMI 52; the Stabilization of Plaques Using Darapladib-Thrombolysis in Myocardial Infarction 52) aiming to determine whether the inhi-bition of plasma Lp-PLA_2_ with its specific inhibitor darapladib has any clinical benefit in the primary and secondary prevention of cardiovascular disease, were negative^[74- 75]^ . Since the inhibitory effect of darapladib on Lp-PLA_2_ has been studied in total plasma and on LDL^[76]^, it is not known whether and to which extent darapladib inhibits Lp-PLA_2_ associated with the anti-atherogenic HDL as well as with Lp(a). Thus, we may speculate that any possible inhibitory effect of darap-ladib on circulating Lp(a)-Lp-PLA_2_ may represent an adverse effect of this agent, thus partially explaining its failure to show a clinical benefit in the above men-tioned phase 3 clinical trials.

### Conclusions and perspectives

OxPL may significantly contribute or even pri-marily account for the atherogenicity of Lp(a) and its increased risk for cardiovascular disease. OxPL may be also significantly involved in the causal role that Lp(a) plays a in the pathophysiology of CAVS. In addition, abnormal function of Lp(a)-Lp-PLA_2_ may significantly influence Lp(a) functionality. However, further studies are required to assess the coordinated role of OxPL and Lp-PLA_2_ on Lp(a) as well as their pathophysiological and clinical relevance in large data sets. Furthermore, the hypothesis drawn in the present review for a patho-genic role of Lp(a) at high concentrations driven mainly by its OxPL and Lp-PLA_2_ content should be established by clinical studies using specific Lp(a) lowering ther-apies. Up to date, there are data from new therapies that reduce Lp(a), such as the cholesteryl ester trans-fer protein inhibitors^[77]^, the antisense oligonucleotide mipomersen^[78]^, and the proprotein convertase subtili-sin/kexin type 9 (PCSK9) inhibitors^[79]^; however, such therapies influence other lipid components in tandem. Thus, from the results of studies evaluating the above drugs, it is not possible to draw safe conclusions on the clinical importance for reducing Lp(a) as well as for the pathophysiological role of this lipoprotein and especially of its OxPL and Lp-PLA_2_ content. Recently, the pharmacokinetics, pharmacodynamics and safety of a second-generation antisense drug (ISIS-APO(a) Rx) designed to reduce the synthesis of apo(a) in the liver were evaluated in a randomised, double-blind, placebo-controlled, phase 1 study performed in healthy adults with plasma Lp(a) levels≥25 nmol/L (100 mg/L). The results showed that ISIS-APO(a)Rx, selectively and potently reduces plasma Lp(a) concentrations as well as OxPL/apoB and OxPL/apo(a) levels in a dose dependent manner without affecting the plasma levels of other lipoproteins^[80]^. No serious or severe adverse events were recorded^[80]^. These results provide the basis for future clinical trials to test whether lowering Lp(a) plasma concentrations and specifically influencing the Lp(a)-Lp-PLA_2_-OxPL axis will reduce the risk of CVD and CAVS.

## References

[1] Hobbs HH, White AL. Lipoprotein: intrigues and insights [J]. , 1999, 10(3): 225–236. 10.1097/00041433-199906000-00005 10431659

[2] Scanu AM, Nakajima K, Edelstein C. Apolipoprotein (a): structure and biology[J]. , 2001, 6: D546–D554. 10.2741/scanu 11229878

[3] Utermann, G. The mysteries of lipoprotein(a)[J[J].]. , 1989, 246(4932): 904–910. 10.1126/science.2530631 2530631

[4] Kostner KM, Kostner GM. Lipoprotein(a): still an enig-ma?[J]. , 2002, 13(4): 391–396. 10.1097/00041433-200208000-00006 12151854

[5] Berglund L, Ramakrishnan R. Lipoprotein(a): An elusive cardiovascular risk factor[J]. , 2004, 24(12): 2219–2226. 10.1161/01.ATV.0000144010.55563.63 PMC3275911 15345512

[6] Gabel BR, Koschinsky MI. Analysis of the proteolytic activity of a recombinant form of apolipoprotein(a)[J]. , 1995, 34(48): 15777–15784. 10.1021/bi00048a023 7495809

[7] McLean JW, Tomlinson JE, Kuang WJ, cDNA sequence of human apolipoprotein(a) is homologous to plasminogen[J]. , 1987, 330(6144): 132–137. 10.1038/330132a0 3670400

[8] Dubé, JB, Boffa MB, Hegele RA, Koschinsky ML. Lipoprotein(a): more interesting than ever after 50 years[J]. , 2012, 23(2): 133–140. 10.1097/MOL.0b013e32835111d8 22327610

[9] Kronenberg, F, Utermann G. Lipoprotein(a): resurrected by genetics[J]. , 2013, 273(1): 6–30. 10.1111/j.1365-2796.2012.02592.x 22998429

[10] Hoover-Plow J, Huang M. Lipoprotein(a) metabolism: potential sites for therapeutic targets[J]. , 2013, 62(4): 479–491. 10.1016/j.metabol.2012.07.024 PMC3547132 23040268

[11] Utermann G. Lipoprotein(a)[J]. In: Scriver CR, Beaudet AL, Sly WS, Valle D, eds. The Metabolic and Molecular Bases of Inherited Disease. New York, NY: McGraw-Hill, Medical Publishing Division. 2006: 2753–87.

[12] Clarke R, Peden JF, Hopewell JC, Genetic variants associated with Lp(a) lipoprotein level and coronary dis-ease[J]. , 2009, 361(26): 2518–2528. 10.1056/NEJMoa0902604 20032323

[13] Tsimikas S, Hall JH. Lipoprotein(a) as a potential causal genetic risk factor of cardiovascular disease: a rationale for increased efforts to understand its pathophysiology and develop targeted therapies[J]. , 2012, 60(8): 716–721. 10.1016/j.jacc.2012.04.038 22898069

[14] Tsimikas, S, Brilakis ES, Miller ER, Oxidized phos-pholipids, Lp(a) lipoprotein, and coronary artery dis-ease[J]. , 2005, 353(1): 46–57. 10.1056/NEJMoa043175 16000355

[15] Bochkov VN, Oskolkova OV, Birukov KG, Generation and Biological Activities of Oxidized Phospholipids[J]. , 2010, 12(8): 1009–1059. 10.1089/ars.2009.2597 PMC3121779 19686040

[16] Bochkov VN. Inflammatory profile of oxidized phospho-lipids[J]. , 2007, 97(3): 348–354. 17334500

[17] Leitinger N. Oxidized phospholipids as modulators of inflammation in atherosclerosis[J]. , 2003, 14(5): 421–430. 10.1097/00041433-200310000-00002 14501580

[18] Watson AD, Berliner JA, Hama SY, Protective effect of high density lipoprotein associated paraoxonase: inhibition of the biological activity of minimally oxidized low density lipoprotein[J]. , 1995, 96(6): 2882–2891. 10.1172/JCI118359 PMC185999 8675659

[19] Watson AD, Navab M, Hama SY, Effect of plate-let activating factor-acetylhydrolase on the formation and action of minimally oxidized low density lipoprotein[J]. , 1995, 95(2): 774–782. 10.1172/JCI117726 PMC295551 7860760

[20] Watson AD, Subbanagounder G, Welsbie DS, Structural identification of a novel pro-inflammatory epoxyisoprostane phospholipid in mildly oxidized low density lipoprotein[J]. , 1999, 274(35): 24787–2479 10.1074/jbc.274.35.24787 10455151

[21] Watson AD, Leitinger N, Navab M, Structural iden-tification by mass spectrometry of oxidized phospholip-ids in minimally oxidized low density lipoprotein that induce monocyte/endothelial interactions and evidence for their presence in vivo[J]. , 1997, 272(21): 13597–13607. 10.1074/jbc.272.21.13597 9153208

[22] Tsimikas S, Bergmark C, Beyer RW, Temporal increases in plasma markers of oxidized low-density lipo-protein strongly reflect the presence of acute coronary syn-dromes[J]. , 2003, 41(3): 360–370. 10.1016/s0735-1097(02)02769-9 12575961

[23] Tsimikas S, Lau HK, Han K-R, Percutaneous Coronary Intervention Results in Acute Increases in Oxidized Phospholipids and Lipoprotein(a). Short-Term and Long-Term Immunologic Responses to Oxidized Low-Density Lipoprotein[J]. , 2004, 109(25): 3164–3170. 10.1161/01.CIR.0000130844.01174.55 15184281

[24] Friedman P, Horkko S, Steinberg D, Correlation of antiphospholipid antibody recognition with the structure of synthetic oxidized phospholipids. Importance of Schiff base formation and aldol condensation[J]. , 2002, 277(9): 7010–7020. 10.1074/jbc.M108860200 11744722

[25] Bergmark C, Dewan A, Orsoni A, A novel function of lipoprotein [a] as a preferential carrier of oxidized phos-pholipids in human plasma[J]. , 2008, 49(10): 2230–2239. 10.1194/jlr.M800174-JLR200 18594118

[26] Leibundgut G, Scipione C, Yin H, Determinants of binding of oxidized phospholipids on apolipopro-tein (a) and lipoprotein (a)[J]. , 2013, 54(10): 2815–2830. 10.1194/jlr.M040733 PMC3770094 23828779

[27] Kochl S, Fresser F, Lobentanz E, Novel interaction of apolipoprotein(a) with β-2 glycoprotein I mediated by the kringle IV domain[J]. , 1997, 90(4): 1482–1489. 9269765

[28] McNeil PH, Simpson RJ, Chesterman CN, Krilis SA. Antiphospholipid antibodies are directed against a com-plex antigen that includes a lipid-binding inhibitor of coag-ulation: β2-Glycoprotein I (apolipoprotein H)[J]. , 1990, 87(11): 4120–4124. 10.1073/pnas.87.11.4120 PMC54059 2349221

[29] Hasunuma Y, Matsuura E, Makita Z, Involvement of beta 2-glycoprotein I and anticardiolipin antibodies in oxidatively modified low-density lipoprotein uptake by macrophages[J]. , 1997, 107(3): 569–73. 10.1046/j.1365-2249.1997.d01-948.x 9067534

[30] Tsironis LD, Katsouras CS, Lourida ES, Reduced PAF-acetylhydrolase activity associated with Lp(a) in patients with coronary artery disease[J]. , 2004, 177(1): 193–201. 10.1016/j.atherosclerosis.2004.07.030 15488884

[31] Zimman A, Mouillesseaux KP, Le T, Vascular endothe-lial growth factor receptor 2 plays a role in the activation of aortic endothelial cells by oxidized phospholipids[J]. , 2007, 27(2): 332–338. 10.1161/01.ATV.0000252842.57585.df 17110601

[32] Subbanagounder G, Wong JW, Lee H, Epoxyisoprostane and epoxycyclopentenone phospholip-ids regulate monocyte chemotactic protein-1 and interleu-kin-8 synthesis. Formation of these oxidized phospholipids in response to interleukin-1 beta[J]. , 2002, 277(9): 7271–7281. 10.1074/jbc.M107602200 11751881

[33] Furnkranz A, Schober A, Bochkov VN, Oxidized phospholipids trigger atherogenic inflammation in murine arteries[J]. , 2005, 25(3): 633–638. 10.1161/01.ATV.0000153106.03644.a0 15591214

[34] Lee H, Shi W, Tontonoz P, Role for peroxisome pro-liferator-activated receptor alpha in oxidized phospholip-id-induced synthesis of monocyte chemotactic protein-1 and interleukin-8 by endothelial cells[J]. , 2000, 87(6): 516–521. 10.1161/01.res.87.6.516 10988245

[35] Reddy ST, Grijalva V, Ng C, Identification of genes induced by oxidized phospholipids in human aortic endothelial cells[J]. , 2002, 38(4): 211–218. 10.1016/s1537-1891(02)00171-4 12449017

[36] Kadl A, Huber J, Gruber F, Analysis of inflamma-tory gene induction by oxidized phospholipids *in vivo* by quantitative real-time RT-PCR in comparison with effects of LPS[J] [J]. , 2002, 38(4): 219–227. 10.1016/s1537-1891(02)00172-6 12449018

[37] Gargalovic PS, Gharavi NM, Clark MJ, The unfolded protein response is an important regulator of inflammatory genes in endothelial cells[J]. , 2006, 26(11): 2490–2496. 10.1161/01.ATV.0000242903.41158.a1 16931790

[38] Huo Y, Weber C, Forlow SB, The chemokine KC, but not monocyte chemoattractant protein-1, triggers mono-cyte arrest on early atherosclerotic endothelium[J]. , 2001, 108(9): 1307–1314. 10.1172/JCI12877 PMC209441 11696575

[39] Berliner JA, Gharavi NM. Endothelial cell regulation by phospholipid oxidation products[J]. , 2008, 45(2): 119–123. 10.1016/j.freeradbiomed.2008.04.013 PMC2895487 18460347

[40] Gargalovic PS, Imura M, Zhang B, Identification of imflammatory gene modules based on variations of human endothelial cell responses to oxidized lipids[J]. , 2006, 103(34): 12741–12746. 10.1073/pnas.0605457103 PMC1568918 16912112

[41] Huber J, Valves A, Mitulovic G, Oxidized membrane vesicles and blebs from apoptotic cells contain biologi-cally active oxidized phospholipids that induce monocyte endothelial interactions[J]. , 2002, 22(1): 101–107. 10.1161/hq0102.101525 11788468

[42] Chen R, Yang L, MsIntyre TM. Cytotoxic phospholipid oxidation products. Cell death from mitochondrial damage and the intrinsic caspase cascade[J]. , 2007, 282(34): 24842–24850. 10.1074/jbc.M702865200 PMC2701377 17597068

[43] Subbanagounder G, Leitinger N, Schwenke DC, Determinants of bioactivity of oxidized phospholipids. Specific oxidized fatty acyl groups at the sn-2 position[J]. , 2000, 20(10): 2248–2254. 10.1161/01.atv.20.10.2248 11031211

[44] Podrez EA, Poliakov E, Shen Z, A novel family of atherogenic oxidized phospholipids promotes macrophage foam cell formation via the scavenger receptor CD36 and is enriched in atherosclerotic lesions[J]. , 2002, 277(41): 38517–38523. 10.1074/jbc.M205924200 12145296

[45] Taleb A, Witztum JL, Tsimikas S. Oxidized phospholipids on apoB-100-containing lipoproteins: a biomarker predict-ing cardiovascular disease and cardiovascular events[J]. , 2011, 5(5): 673–694. 10.2217/bmm.11.60 PMC3230643 22003918

[46] Kiechl S, Willeit J, Mayr M, Oxidized phospholipids, lipoprotein(a), lipoprotein-associated phospholipase A2 activity, and 10-year cardiovascular outcomes: prospective results from the Bruneck study[J]. , 2007, 27(8): 1788–1795. 10.1161/ATVBAHA.107.145805 17541022

[47] Tsimikas S, Willeit P, Willeit J, Oxidation-specific biomarkers, prospective 15-year cardiovascular and stroke outcomes, and net reclassification of cardiovascular events[J]. , 2012, 60(21): 2218–2229. 10.1016/j.jacc.2012.08.979 23122790

[48] Erqou S, Thompson A, Di Angelantonio E, , Apolipoprotein(a) Isoforms and the Risk of Vascular Disease. Systematic Review of 40 Studies Involving 58,000 Participants[J]. , 2010; 55(19): 2160–2167. 10.1016/j.jacc.2009.10.080 20447543

[49] Rajamannan NM, Evans FJ, Aikawa E, Calcific aortic valve disease: not simply a degenerative process: a review and agenda for research from the National Heart and Lung and Blood Institute Aortic Stenosis Working Group. Executive summary: calcific aortic valve disease-2011 update[J]. , 2011, 124(16): 1783–1791. 10.1161/CIRCULATIONAHA.110.006767 PMC3306614 22007101

[50] Thanassoulis G, Campbell CY, Owens DS, Genetic associations with valvular calcification and aortic steno-sis[J]. , 2013, 368(6): 503–512. 10.1056/NEJMoa1109034 PMC3766627 23388002

[51] Kamstrup PR, Tybjærg-Hansen A, Nordestgaard BG. Elevated Lipoprotein(a) and Risk of Aortic Valve Stenosis in the General Population[J]. , 2014, 63(5): 470–477. 10.1016/j.jacc.2013.09.038 24161338

[52] Capoulade R, Chan KL, Yeang C, Oxidized Phospholipids, Lipoprotein(a), and Progression of Calcific Aortic Valve Stenosis[J]. , 2015, 66(11): 1236–1246. 10.1016/j.jacc.2015.07.020 26361154

[53] Hung M-Y, Witztum JL, Tsimikas S. New Therapeutic Targets for Calcific Aortic Valve Stenosis. The Lipoprotein(a)-Lipoprotein-Associated Phospholipase A2-Oxidized Phospholipid Axis[J]. , 2014, 63(5): 478–480. 10.1016/j.jacc.2013.08.1639 PMC5928500 24161316

[54] Jira W, Spiteller G, Richter A. Increased levels of lipid oxidation products in low density lipoproteins of patients suffering from rheumatoid arthritis[J]. , 1997, 87(1): 81–89. 10.1016/s0009-3084(97)00030-3 9219348

[55] Savaskan NE, Ufer C, Kuhn H, Borchert A. Molecular biology of glutathione peroxidase 4: From genomic struc-ture to developmental expression and neural function[J]. 2007, 388(10): 1007–1017. 10.1515/BC.2007.126 17937614

[56] Jin Y, Penning TM. Aldo-keto reductases and bioactiva-tion/detoxication[J]. , 2007, 47: 263–292. 10.1146/annurev.pharmtox.47.120505.105337 16970545

[57] Tselepis AD, Chapman MJ. Inflammation, bioactive lipids and atherosclerosis: potential roles of a lipoprotein-associ-ated phospholipase A2, platelet activating factor-acetylhy-drolase[J]. , 2002, Suppl 3(4): 57–68. 10.1016/s1567-5688(02)00045-4 12573364

[58] Tellis CC, Tselepis AD. The role of lipoprotein-associ-ated phospholipase A2 in atherosclerosis may depend on its lipoprotein carrier in plasma[J]. , 2009, 1791: 327–38. 10.1016/j.bbalip.2009.02.015 19272461

[59] Karabina SA, Elisaf MC, Goudevenos J, PAF-acetylhydrolase activity of Lp(a) before and during Cu(2+)-induced oxidative modification in vitro[J]. , 1996, 125(1): 121–134. 10.1016/0021-9150(96)05872-8 8831934

[60] Blencowe C, Hermetter A, Kostner GM, Deingner HP. Enhanced association of platelet-activating factor acetylhy-drolase with lipoprotein (a) in comparison with low density lipoprotein[J]. , 1995, 270(52): 31151–31157. 10.1074/jbc.270.52.31151 8537378

[61] Stafforini DM, Tjoelker LW, McCormick SP, Molecular basis of the interaction between plasma plate-let-activating factor acetylhydrolase and low density lipo-protein[J]. , 1999, 274(11): 7018–7024. 10.1074/jbc.274.11.7018 10066756

[62] Gardner AA, Reichert EC, Topham MK, Stafforini DM. Identification of a domain that mediates association of platelet-activating factor acetylhydrolase with high density lipoprotein[J]. , 2008, 283(25): 17099–17106. 10.1074/jbc.M802394200 PMC2427361 18434304

[63] Cao J, Hsu YH, Li S, Structural basis of specific interactions of Lp-PLA2 with HDL revealed by hydrogen deuterium exchange mass spectrometry[J]. , 2013, 54(1): 127–133. 10.1194/jlr.M030221 PMC3520519 23089916

[64] Moutzouri E, Tsimihodimos V, Tselepis AD. Inflammatory biomarkers and cardiovascular risk assessment. Current knowledge and future perspectives[J]. , 2013, 19(21): 3827–3840. 10.2174/13816128113199990307 23286437

[65] Tellis CC, Tselepis AD. Pathophysiological role and clinical significance of lipoprotein-associated phospholipase A (Lp-PLA ) bound to LDL and HDL[J]. , 2014, 20(40): 6256–6269. 10.2174/1381612820666140622200916 24953389

[66] Rallidis LS, Tellis CC, Lekakis J, Lipoprotein-associated phospholipase A(2) bound on high-density lipoprotein is associated with lower risk for cardiac death in stable coronary artery disease patients: a 3-year fol-low-up[J]. , 2012, 60(20): 2053–2060. 10.1016/j.jacc.2012.06.057 23083783

[67] Cushing GL, Gaubatz JW, Nava ML, Quantitation and localization of apolipoproteins [a] and B in coro-nary artery bypass vein grafts resected at re-operation[J]. , 1989, 9(5): 593–603. 10.1161/01.atv.9.5.593 2789507

[68] Pepin JM, O’Neil JA, Hoff HF. Quantification of apo (a) and apoB in human atherosclerotic lesions[J]. , 1991, 32(2): 317–327. 1829751

[69] Berliner JA, Subbanagounder G, Leitinger N, Evidence for a role of phospholipid oxidation products in atherogenesis[J]. , 2001; 11(3-4): 142–147. 10.1016/s1050-1738(01)00098-6 11686004

[70] Wei DH, Zhang XL, Wang R, Oxidized lipoprotein(a) increases endothelial cell monolayer permeability via ROS generation[J]. , 2013, 48(6): 579–586. 10.1007/s11745-013-3795-1 23674170

[71] Morishita R, Ishii J, Kusumi Y, Association of serum oxidized lipoprotein(a) concentration with coronary artery disease: potential role of oxidized lipoprotein(a) in the vas-cular wall[J]. , 2009, 16(4): 410–418. 10.5551/jat.no224 19672030

[72] Tsimikas S, Tsironis LD, Tselepis AD. New insights into the role of lipoprotein(a)-associated lipoprotein-associated phospholipase A2 in atherosclerosis and cardiovascular disease[J]. , 2007, 27(10): 2094–2099. 10.1161/01.ATV.0000280571.28102.d4 17626905

[73] Kim YL, Im YJ Ha NC, Im DS. Albumin inhibits cytotoxic activity of lysophosphatidylcholine by direct binding[J]. . 2007, 83(1-2): 130–138. 10.1016/j.prostaglandins.2006.10.006 17259079

[74] STABILITY Investigators, White HD, Held C, Stewart R, Darapladib for preventing ischemic events in stable coronary heart disease[J]. , 2014, 370(18): 1702–1711. 10.1056/NEJMoa1315878 24678955

[75] O’Donoghue ML, Braunwald E, White HD, Effect of darapladib on major coronary events after an acute coro-nary syndrome: the SOLID-TIMI 52 randomized clinical trial[J]. , 2014, 312(10):1006–1015. 10.1001/jama.2014.11061 25173516

[76] Mohler ER 3rd, Ballantyne CM, Davidson MH, The effect of darapladib on plasma lipoprotein-associated phospholipase A2 activity and cardiovascular biomarkers in patients with stable coronary heart disease or coronary heart disease risk equivalent: the results of a multicenter, randomized, double-blind, placebo-controlled study[J]. , 2008, 51(17): 1632–1641. 10.1016/j.jacc.2007.11.079 18436114

[77] Bochem AE, Kuivenhoven JA, Stroes ES. The promise of cholesteryl ester transfer protein (CETP) inhibition in the treatment of cardiovascular disease[J]. , 2013, 19(17): 3143–3149. 10.2174/1381612811319170022 23317399

[78] Santos RD, Raal FJ, Catapano AL, Mipomersen, an antisense oligonucleotide to apolipoprotein B-100, reduces lipoprotein(a) in various populations with hypercholester-olemia: results of 4 phase III trials[J]. , 2015, 35(3): 689–699. 10.1161/ATVBAHA.114.304549 PMC4344404 25614280

[79] Raal FJ, Giugliano RP, Sabatine MS, Reduction in lipoprotein(a) with PCSK9 monoclonal antibody evolo-cumab (AMG 145): a pooled analysis of more than 1,300 patients in 4 phase II trials[J]. , 2014, 63(13): 1278–1288. 10.1016/j.jacc.2014.01.006 24509273

[80] Tsimikas S, Viney NJ, Hughes SG, Antisense therapy targeting apolipoprotein(a): a randomised, double-blind, placebo-controlled phase 1 study[J]. , 2015, 386(10002): 1472–1483. 10.1016/S0140-6736(15)61252-1 26210642

